# A dual-quenched ECL immunosensor for ultrasensitive detection of retinol binding protein 4 based on luminol@AuPt/ZIF-67 and MnO_2_@CNTs

**DOI:** 10.1186/s12951-021-01020-1

**Published:** 2021-09-08

**Authors:** Wei Gong, Suqing Yang, Fen Zhang, Fengshun Tian, Junman Chen, Zhigang Yin, Shijia Ding, Wei Yang, Rong Luo

**Affiliations:** 1 Medical Examination Centre, The First Affiliated Hospital of Chongqing Medical University, Chongqing, 400016 China; 2 Department of Endocrinology, The First Affiliated Hospital of Chongqing Medical University, Chongqing, 400016 China; 3 Chongqing Testing & Lnspection Center for Medical Devices, Chongqing, 400016 China; 4 Key Laboratory of Clinical Laboratory Diagnostics (Ministry of Education), College of Laboratory Medicine, Chongqing Medical University, Chongqing, 400016 China

**Keywords:** Luminol, Zeolitic imidazolate framework-67, MnO_2_ nanosheets, Electrochemiluminescence resonance energy transfer, Retinol binding protein 4

## Abstract

**Background:**

Retinol binding protein 4 (RBP4) has been regarded as an important serological biomarker for type 2 diabetes mellitus (T2DM). Hence, the construction of a highly sensitive detection method for RBP4 is the key to early prevention and multidisciplinary intervention of T2DM. In this work, a dual-quenched electrochemiluminescence (ECL) immunosensor has been fabricated for ultrasensitive detection of RBP4 by combining zeolitic imidazolate framework-67/AuPt-supported luminol (luminol@AuPt/ZIF-67) with MnO_2_ nanosheets-grown on carbon nanotubes (MnO_2_@CNTs).

**Results:**

AuPt/ZIF-67 hybrids with high-efficiency peroxidase-like activity could provide multipoint binding sites for luminol and antibodies and significantly boost the amplified initial signal of the ECL immunosensor. Upon glutathione/H_2_O_2_ coreactants system, MnO_2_@CNTs composites could quench the initial signal by inhibiting mimic peroxidase activity of luminol@AuPt/ZIF-67. Moreover, the absorption spectrum of the MnO_2_@CNTs composites completely overlaps with the emission spectrum of luminol, which can further reduce initial signal by ECL resonance energy transfer (ECL-RET).

**Conclusions:**

Benefiting from the above-mentioned properties, the designed immunoassay sensitivity exhibited excellent sensitivity and relative stability for RBP4 detection range from 0.0001 to 100 ng mL^−1^ with a low detection limit of 43 fg mL^−1^. Therefore, our ECL immunosensor provides an alternative assaying strategy for early diagnosis of T2DM.

**Graphic abstract:**

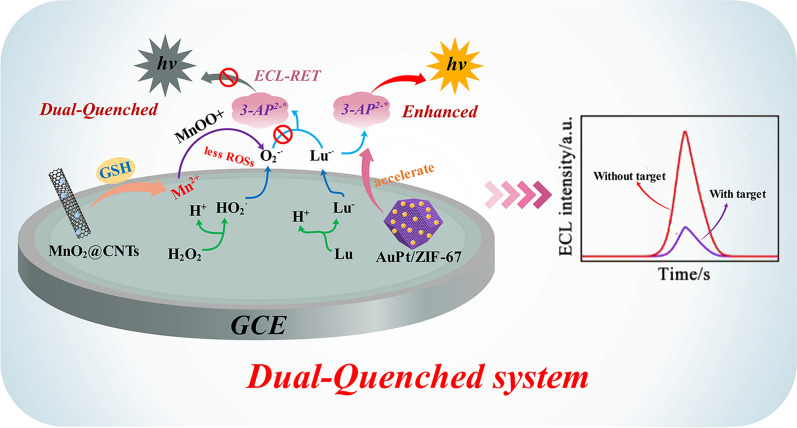

**Supplementary Information:**

The online version contains supplementary material available at 10.1186/s12951-021-01020-1.

## Background

Type 2 diabetes mellitus (T2DM) is a pyramidally widespread multifactor disease with severe life-threatening complications including cardiovascular disease (CVD), heart failure, and chronic kidney disease (CKD) [1, 2]. According to *Global Report on Diabetes* of WHO in 2016 and *The Berlin Declaration* in 2018, early detection and proper multidisciplinary interventions are the priority among priorities of T2DM [3, 4]. Retinol binding protein 4 (RBP4), an insulin resistance-related hormone, is a potential serological biomarker for early monitoring and diagnosis of T2DM [5–7]. However, conventional RBP4 quantitative detection methods, such as enzyme immunoassay (EIA) [8], enzyme linked immunosorbent assay (ELISA) [9], and radioimmunoassay [10], usually suffer from insufficient sensitivity, expensive experimental facilities and complicated operation procedures. Thus, in order to address the aforementioned challenges, establishing effective RBP4 analysis strategy with the advantages of easy manipulation, high sensitivity, and low cost remains urgent requirement for modern clinical diagnosis of T2DM.

Metal organic frameworks (MOFs) as a novel kind of nanomaterial-based artificial enzymes, possess superior biocompatibility, high catalytic activity and durability which demonstrate great potential to replace biological enzymes as high-efficiency catalysts [11–13]. Zeolitic imidazolate frameworks (ZIFs), an attractive subclass of MOFs, have received considerable attention in the bio-analysis applications ascribing to their unique characteristics of large specific surface area, adjustable structure and ordered crystalline pores [14]. More importantly, the imidazolate linkers, especially the imidazole nitrogen site within the porous frameworks, endow ZIFs with excellent selective adsorption properties [15] and catalytic activities [16], making them good candidates in the field of electrochemical sensing.

Furthermore, multi-layered MOFs nanozymes, especially coordinating MOFs with noble metal (Au and Pt) nanoparticles (NPs) demonstrate several significant advantages [17]. For instance, the incorporation of highly dispersed Pt NPs with modulated electronic configuration on a surfactant-free MOF surface has been used to deliver decent electrocatalytic activity under both alkaline and acidic circumstances and promote the electrochemical hydrogen evolution reaction (HER) [18]. More importantly, the introduction of noble metal NPs also can be employed as powerful support matrixes to anchor more molecular luminophores (luminol or its derivatives) by covalent bonding, thus improving the analytical performance of electrochemiluminescence (ECL) biosensor [19, 20]. Inspired by the above research, ZIF-67-supported AuPt bimetallic NPs (AuPt NPs) and luminol nanozymes (luminol@AuPt/ZIF-67) have been synthesized for the first time. By combining the merits of ZIF-67 and AuPt NPs, this composite material not only ensures uniform dispersion of AuPt NPs and luminol but also provide larger surface area to load more antibodies, thus greatly enhancing luminescence efficiency through peroxidase-like activity of ZIF-67.

Additionally, seeking highly efficient quenchers to quench the luminophores is another crucial challenge in ECL immunoassay. The two-dimensional (2D) manganese dioxide (MnO_2_) nanosheets possess high energy density, ecological friendliness and non-toxic properties, which are regarded as one of the most promising high-performance ECL quenchers [21, 22]. Zhang’s group utilized large Pt NPs as the nanocarrier for MnO_2_ nanosheets to realize quantitative detection of glutathione (GSH), in which MnO_2_ nanosheets could react with GSH to generate Mn^2+^, thus scavenging reactive oxygen species (ROSs) properties and inhibiting mimic peroxidase activity of nanozymes [23, 24]. Moreover, the emission spectrum of luminol (~ 425 nm) [25] overlaps the absorption spectrum of MnO_2_ nanosheets (300–600 nm) [26] completely, enabling the efficient ECL-resonance energy-transfer (ECL-RET) to diminish ECL intensities of luminol [27]. This dual-quenching property endows MnO_2_ with superior performance, which is however, also relate to its size and dispersion [28, 29]. Hence other nanomaterials are commonly used to modify MnO_2_ to enhance their catalytic ability as quenchers. Abundant works have reported that carbon nanotubes (CNTs) with various extraordinary properties including unique nano-size, high specific surface area, superior adsorption effect and multifunctional binding sites, have been generally developed as supporting nanomaterials to modify metals, metal oxides and other materials [30–32]. The excellent modification ability and compatibility of carbon nanotubes for other nanomaterials may significantly improve the electrical conductivity, dense morphology and dispersion of MnO_2_. Therefore, the combination of CNTs and MnO_2_ to construct high-efficiency quenchers is the key to further broaden their application in ECL immunoassay.

**Scheme 1:**
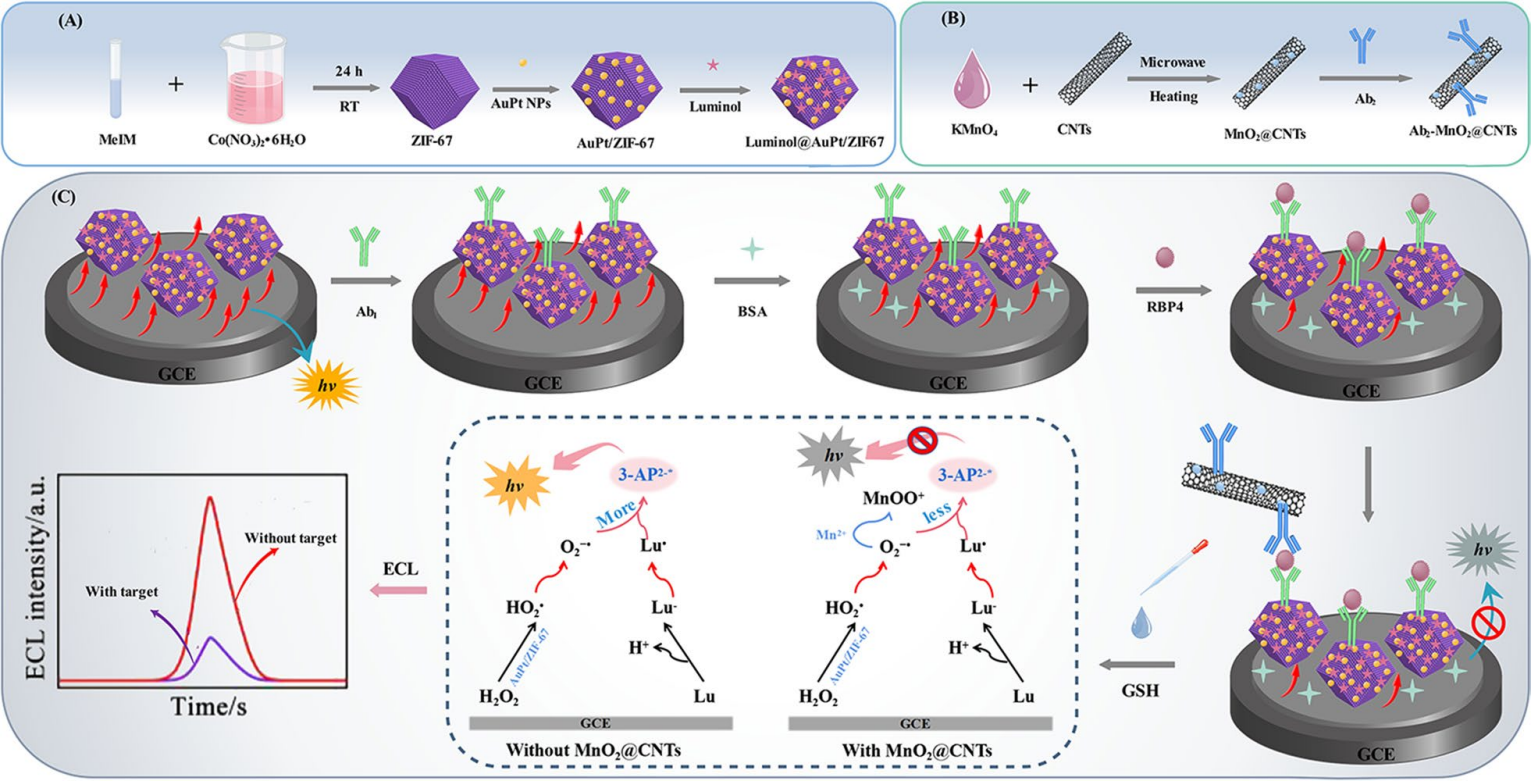
Principle of the dual-quenched ECL immunosensor based on luminol@AuPt/ZIF-67 and MnO_2_@CNTs for the detection of RBP4. **A** The preparation procedure of the luminol@AuPt/ZIF-67. **B** The synthesis route of Ab_2_-MnO_2_@CNTs. **C** The detection process and operation mechanism of the sandwich-type immunosensor

Herein, a dual-quenched ECL-RET immunosensor was established for ultrasensitive detection of RBP4 based on luminol@AuPt/ZIF-67 nanozymes and MnO_2_@CNTs quenchers. As illustrated in Scheme 1A, AuPt/ZIF-67 was stepwise synthesized by a simple wet-chemical method. Subsequently, luminol was combined with AuPt/ZIF-67 through covalent bonding between AuPt NPs and the amino groups of luminol to form luminol@AuPt/ZIF-67 hybrids as ECL donors. As shown in Scheme 1B, by growing 2D MnO_2_ nanosheets on the surface of CNTs, the high-performance MnO_2_@CNTs quenchers are in situ synthesized as the ECL acceptor and then conjugated with RBP4 secondary antibodies (Ab_2_) through electrostatic effect, forming Ab_2_-MnO_2_@CNTs composites. As displayed in Scheme 1C, the ECL immunosensor was prepared by incubating RBP4 monoclonal antibody (Ab_1_) on the glassy carbon electrode (GCE) modified with luminol@AuPt/ZIF-67 hybrids. In the presence of RBP4, the Ab_2_-MnO_2_@CNTs bioconjugates were immobilized on the surface of luminol@AuPt/ZIF-67 decorated electrode based on a sandwich-like immuno response. Meanwhile, GSH was introduced into the system as a reducing agent to constructed a dual-quenched immunosensor according to the ECL-RET and consumption of ROSs by Mn^2+^, which successfully achieved highly sensitive enzyme-free detection of RBP4.

## Methods

### Preparation of luminol@AuPt/ZIF-67 hybrids

ZIF-67 particles was synthesized in accordance with the method reported previously [33]. Typically, 0.364 g of Co(NO_3_)_2_·6H_2_O and 0.41 g of MeIM was dispersed in 20 mL methanol solution, respectively. The former pink solution was added into the colorless solution under vigorous stirring. The mixture was treated with ultrasound for about 1 min and kept static at room temperature (RT) overnight. The sediment with purple color was collected by centrifugation (9000 rpm, 5 min) and then washed with methanol five times.

The AuPt/ZIF-67 hybrids preparation was shown in Scheme 1A. The previously synthesized ZIF-67 was added into 20 mL methanol solution and stirred for 25 min to acquire a uniformly dispersed solution. After that, 0.5 mL of HAuCl_4_·6H_2_O (1.0 wt%) and 0.5 mL of H_2_PtCl_6_·6H_2_O (1.0 wt%) were added to the ZIF-67 solution and stirred for 15 min. Then 1 mL of 1.5 M freshly NaBH_4_ was slowly added into the mixed solution under vigorous stirring for 1.5 h. AuPt/ZIF-67 hybrids were obtained by centrifugation of the mixture with methanol. After that, the resulted AuPt/ZIF-67 was re-suspended in 10 mL methanol solution, then 0.5 mL 10 mM of luminol was added to the AuPt/ZIF-67 solution and fully stirred for 10 h. The mixed solution was centrifuged and washed to clean uncombined luminol. Lastly, the mixtures were dissolved in 1% chitosan solution and preserved at 4 °C for later use.

### *Fabrication of Ab*_*2*_*-MnO*_*2*_*@CNTs bioconjugates*

Firstly, 0.03 g KMnO_4_ was deliquesced in 45 mL deionized water under magnetic stirring. Then 20 mg CNTs, 1 M H_2_SO_4_ (2 mL) and 1 mL PDDA were added to the above suspension and sonicated for 2 h with a time interval of 30 min. CNTs and KMnO_4_ could be better dispersed by this ultrasonic treatment step to promote monomer adsorption on the CNTs wall. After ultrasonic treatment, a household microwave oven (Midea M1-L201B 800 W) was used to heat the mixed solution for 1 min, and then cooled to RT. The heating and cooling processes were repeated 5 times. The precipitate was gathered by centrifuge and cleaning with deionized water several times. The final sediment (MnO_2_@CNTs composites) was dried at 100 °C overnight for later use.

MnO_2_@CNTs and Ab_2_ were coupled by electrostatic effect. 2 mg MnO_2_@CNTs were added to 5 mL PBS (0.01 M, pH 7.4) and stirred for 2 h. After that, 600 µL 10 µg mL^−1^ Ab_2_ was added to the obtained MnO_2_@CNTs solution for mixing and stirred at 4 °C for 12 h. Then, 400 µL 1.0 wt% BSA solution was introduced into above solution and continuously stirred at 4 °C for 1 h to prevent nonspecific binding, followed by centrifuging. The as-prepared Ab_2_-MnO_2_@CNTs bioconjugates were re-suspended in 1 mL PBS (0.01 M, pH 7.4) at 4 °C for further use.

### ECL detection of RBP4

The bare GCE was polished with 0.3 and 0.05 µm alumina (Al_2_O_3_) slurry for 3 min to acquire a mirror-like surface, followed by rinsing thoroughly with deionized water. Then, 5 µL luminol@AuPt/ZIF-67 and Ab_1_ (1 µg mL^−1^) suspension were incubated at 4 °C for least 2 h, then washed with PBS (0.01 M, pH 7.4) to remove unbounded Ab_1_. Next, the electrode was covered with 3 µL 1.0 wt% BSA solution to block the nonspecific binding for 45 min at 4 °C. After rinsing with PBS (0.01 M, pH 7.4) thoroughly, 10 µL RBP4 of different concentrations were pipetted onto the AuPt/ZIF-67 decorated electrode surface then dried at 4 °C for least 1.5 h. Finally, 5 µL Ab_2_-MnO_2_@CNTs bioconjugates was reacted with the modified electrode for 1 h to form the double antibody sandwich complex and further cleaned with PBS (0.01 M, pH 7.4).

## Results and discussion

### Characterization of ZIF-67 and AuPt/ZIF67

**Fig. 1:**
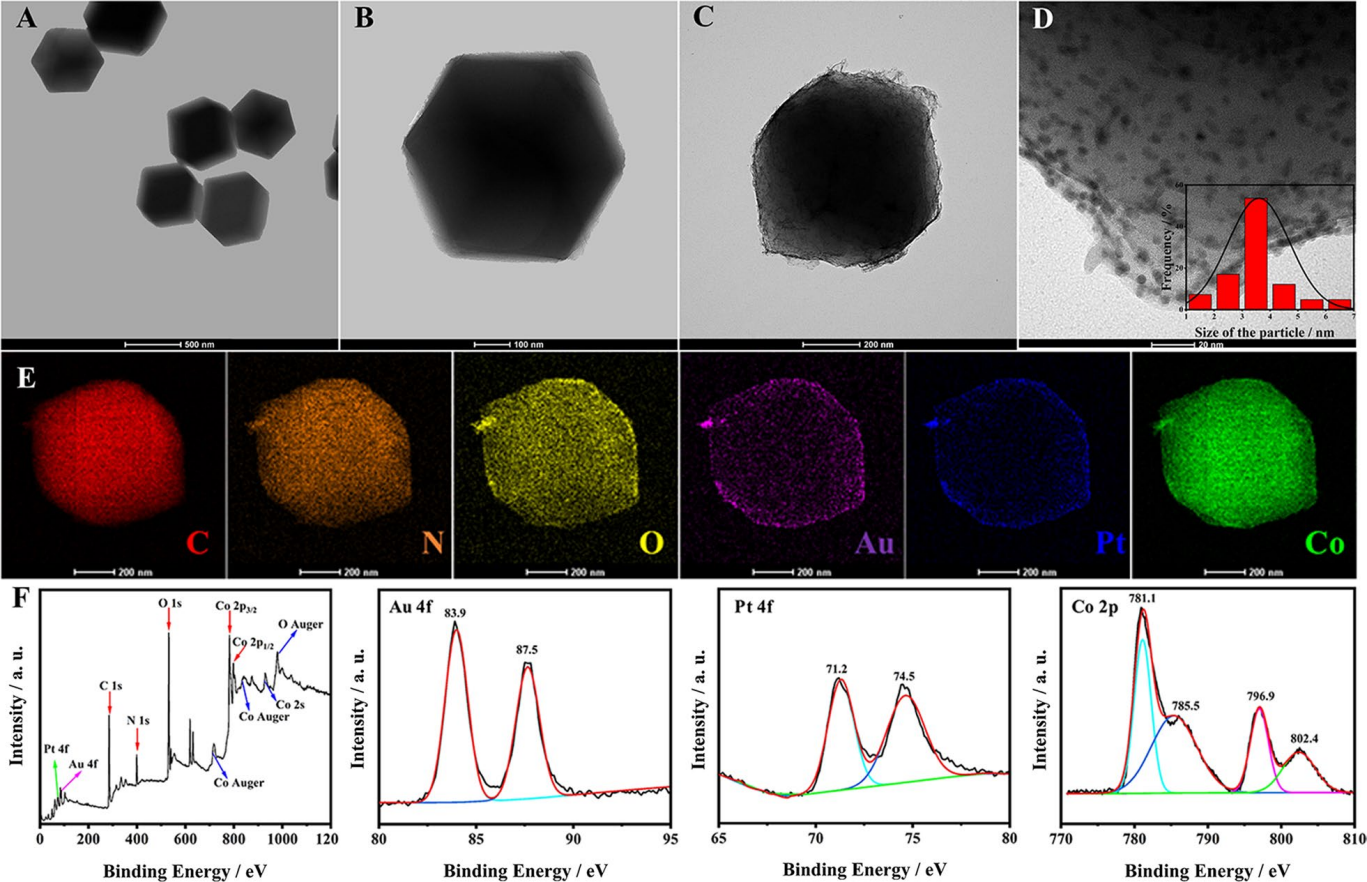
**A** Low-magnifcation and **B** high-magnification TEM image of ZIF-67. **C** Low-magnification and **D** high-magnification TEM image of AuPt/ZIF-67. The inset is the size distribution of AuPt NPs. **E** STEM-EDS mappings of AuPt/ZIF-67. **F** XPS high resolution spectra of AuPt/ZIF-67

The size, morphology and elemental composition of the as-synthesized nanomaterials were characterized by Transmission electron microscopy (TEM) and Scanning transmission electron microscopy-energy dispersive X-ray spectroscopy (STEM-EDS). As illustrated in Fig. 1A, B, the synthesized ZIF-67 showed a dodecahedral framework with a side length of about 500 nm and a very glossily surface. Moreover, after decorating with AuPt NPs, it was clear that the AuPt NPs were homogeneously dispersed on the surface of ZIF-67 (Fig. 1C, D) with an apex-to-apex diameter about 3 nm. The element composition of AuPt/ZIF-67 was manifested by the elemental mappings of C, N, O, Au, Pt, Co via STEM-EDS elemental mapping (Fig. 1E), which further proved that the surface of the ZIF-67 was uniformly dispersed AuPt bimetallic nanoparticles.

X-ray photoelectron spectroscopy (XPS) was used to further research the elemental composition and surface electronic state of the AuPt/ZIF-67 hybrids. As illustrated in Fig. 1F, the survey spectrum of AuPt/ZIF-67 indicated the presence of Au, Pt, C, N, O and Co. The fitted peaks located at 83.9 eV and 87.5 eV were indexed to Au 4f peaks, indicating that the surface of the ZIF-67 formed metallic gold [20]. The fitted peaks of Pt 4f at 71.2 eV and 74.5 eV, demonstrated the forming of metallic Pt [34]. As shown in the high-resolution spectra of Co 2p, the fitted peaks at 781.1 eV, 785.5 eV, 796.9 eV and 802.4 eV could be assigned to metallic Co [35]. The XPS results were consistent with the STEM-EDS mapping results, indicating that the modified AuPt/ZIF-67 composites were successfully synthesized as expected.

To investigate the crystal structure, the ZIF-67 and AuPt/ZIF-67 composites were also characterized using X-ray diffraction (XRD). As seen from Additional file 1: Fig. S1, the diffraction peaks of AuPt/ZIF-67 coincided exactly with simulated ZIF-67, demonstrating that the successful assembly of AuPt NPs on ZIF-67. Furthermore, the absence of XRD peaks from AuPt NPs could be probably attributed to the low NPs concentration as well as the small size of encapsulated AuPt NPs in the ZIF-67 framework.

### *Characterization of CNTs and MnO*_*2*_*@CNTs nanocomposites*

**Fig. 2:**
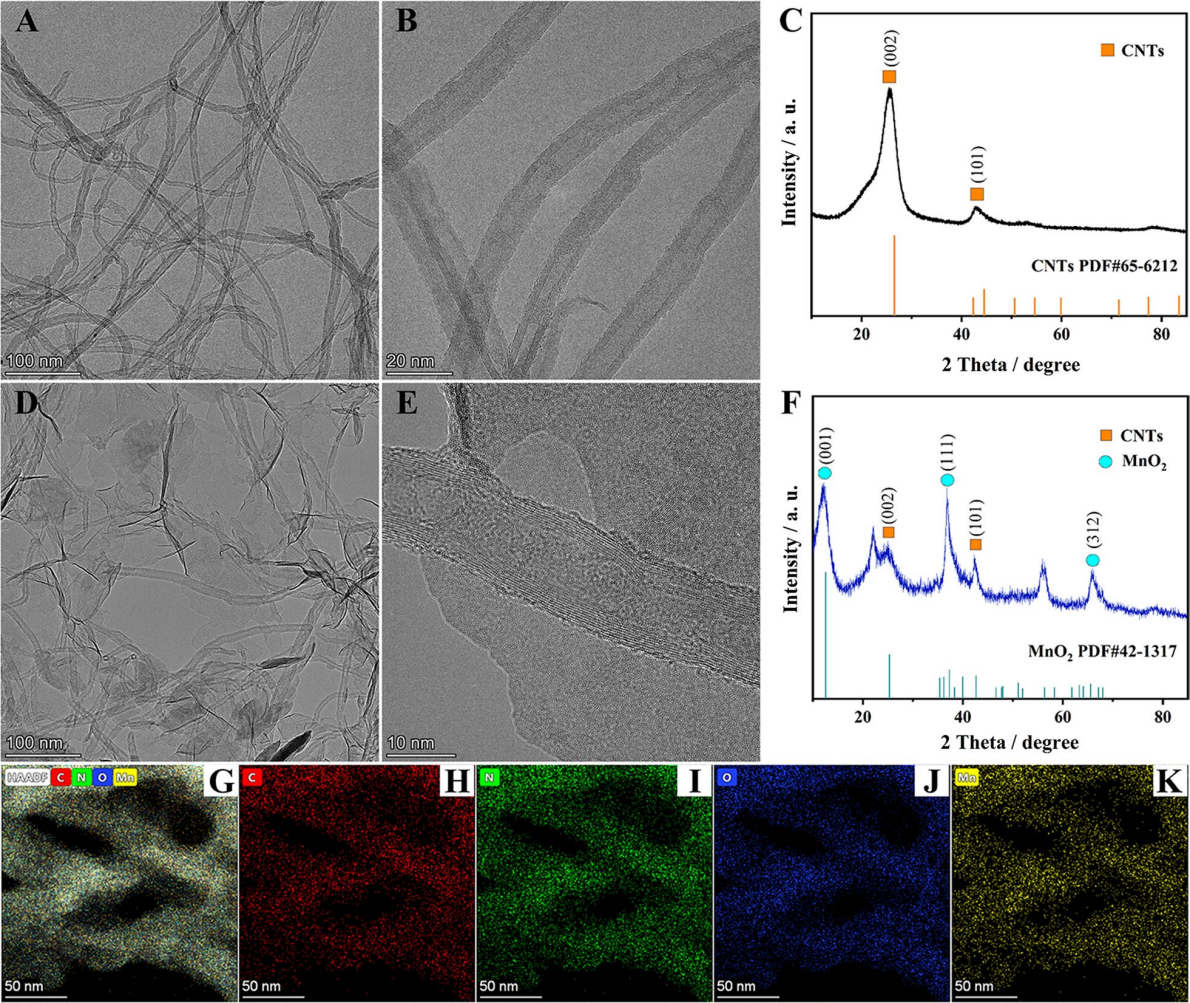
**A** Low-magnification and **B** high-magnification TEM image of CNTs. **D** Low-magnification and **E** high-magnification TEM image of MnO_2_@CNTs. XRD pattern of **C** CNTs and **F** MnO_2_@CNTs. **G** HAADF image and **H**–**K** STEM-EDS elemental mappings of MnO_2_@CNTs composites

The morphology and structure of CNTs and MnO_2_@CNTs were characterized with TEM, high-angle annular dark-field (HAADF) and energy-dispersive X-ray spectroscopy (EDS). From Fig. 2A, B, the as-prepared CNTs were uniform tubular structure with the average diameter about 8 nm. After decorated with MnO_2_, it could be seen that 2D MnO_2_ nanosheets in situ grows on the surface of CNTs with a typical layer-like structure. (Fig. 2D, E). Meanwhile, elemental analysis using EDS mapping was performed in Fig. 2G–K, the occurrence of C, N, O, and Mn elements demonstrated that MnO_2_ nanosheets were uniformly dispersedly bonded onto the surface of CNTs.

The more detailed elemental composition and the oxidation state in the MnO_2_@CNTs were characterized by XPS measurements, and the results of high-resolution XPS spectra of C 1s, O 1s, and Mn 2p are exhibited in Additional file 1: Fig. S2A. As shown in Additional file 1: Fig. S2B, the survey spectrum of C 1s indicated three peaks at 284.3 eV, 285.1 eV, 286.1 eV which consisted of the C=C, C–C, C=O functional groups, respectively [36]. The high-resolution spectra of O 1s in Additional file 1: Fig. S2C displayed two broad peak at 529.6 eV and 531.1 eV, which could be correspond to two oxygen contributions [37]. There were two peaks located at 641.8 eV and 653.2 eV that could be assigned to Mn 2p_3/2_ and Mn 2p_1/2_ in Additional file 1: Fig. S2D. The peak separation of 11.4 eV between Mn 2p_3/2_ and Mn 2p_1/2_ in the MnO_2_@CNTs demonstrated that Mn was present in the composite in the Mn^4+^ state [38].

XRD analysis was carried out to further explore the structure of MnO_2_@CNTs nanocomposite material. As shown in Fig. 2C, two stable diffraction peaks at 26° and 42° (marked with orange labels) indexed to the (002) and (101) crystal plane of CNTs (PDF card: JCPDS 65-6212) were observed. Furthermore, in the XRD pattern of MnO_2_@CNTs (Fig. 2F), there were three broad peaks at around 12°, 37° and 66° (marked with blue labels) could be obviously indexed to the (001), (111) and (312) crystal plane of MnO_2_ (PDF card: JCPDS 42-1317) except for the two diffraction peak of the CNTs pane [39]. All these characterizations demonstrated the successful synthesis of MnO_2_@CNTs.

### Enhancement and dual-quenched mechanism of the ECL immunosensor

**Fig. 3:**
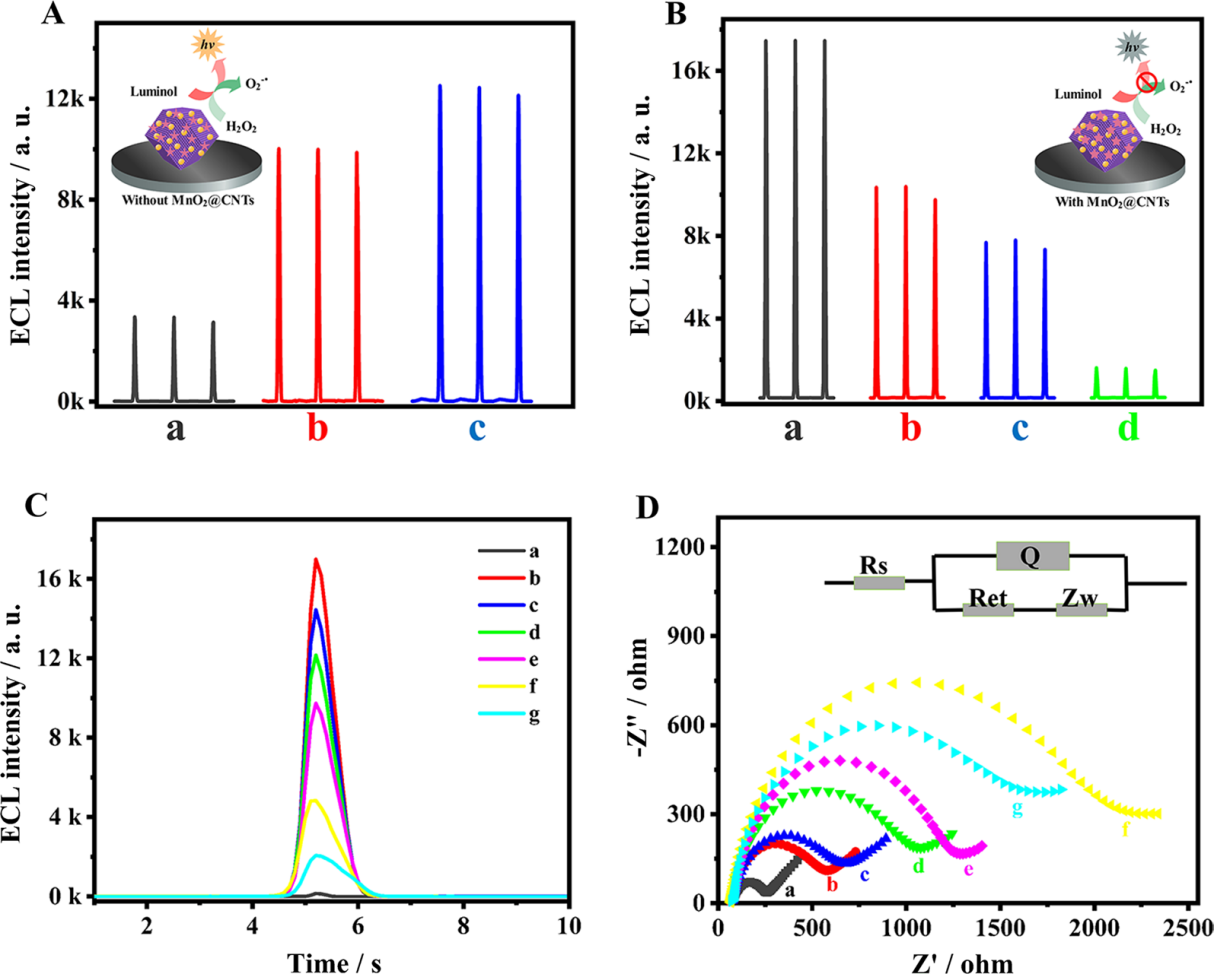
**A** ECL behavior of pure luminol (a), ZIF-67@luminol (b), luminol@AuPt/ZIF-67 (c). **B** ECL behavior of luminol@AuPt/ZIF-67/GCE without (a) and with (b) GSH, MnO_2_@CNTs/luminol@AuPt/ZIF-67/GCE without (c) and with (d) GSH in 5 mL PBS (0.1 M, pH 8.0) containing 10 mM H_2_O_2_. **C** ECL curve and **D** EIS of bare GCE (a), after luminol@AuPt/ZIF-67 modification (b), after Ab_1_ modification (c), after BSA blocking (d), after 10 ng mL^−1^ RBP4 incubation (e), after Ab_2_-MnO_2_@CNTs immobilization (f), and after adding 20 mM GSH (g), respectively measured in 5 mL PBS (0.1 M, pH 8.0) containing 10 mM H_2_O_2_ and in 5 mM Fe(CN)_6_^3−/4−^ containing 0.1 M KCl at 150 mV s^−1^. The inset was the equivalent circuit for EIS

A possible ECL mechanism of the immunosensor was proposed as displayed in Scheme 1C. Luminol was oxidized to luminol anion (Lu^−^) which could transform into intermediate oxidation state of luminol (Lu·) under the scan of a positive potential. The synthesized AuPt/ZIF67 possessed superior peroxidase activity and excellent catalytic effect on H_2_O_2_, thus catalyzing H_2_O_2_ production of superoxide anion (O_2_^−**.**^). Subsequently, Lu· would react with superoxide anion O_2_^−**.**^ to generate the excited state of 3-aminophthalate (3-AP^2−^∗). Then, a heavily ECL signal was generated when the excited state 3-AP^2−^∗ returns to the ground state 3-AP^2−^. In order to demonstrate the successful assembly of luminol on AuPt/ZIF-67 surface, the UV–Vis absorbance spectra of luminol, ZIF-67, AuPt/ZIF-67 and luminol@AuPt/ZIF-67 were monitored (Additional file 1: Fig. S4). The UV–Vis absorbance spectrum of luminol@AuPt/ZIF-67 (curve d) could be observed at 355 nm and 600 nm with two distinct characteristic peaks corresponding to the absorbance of luminol (curve b) and ZIF-67 (curve a), respectively. To explore the ECL enhancement of luminol by the synthesized nanocomposites, we measured the ECL intensities of pure luminol (curve a), luminol@ZIF-67 (curve b) and luminol@AuPt/ZIF-67 (curve c) in solution (Fig. 3A). Compared with pure luminol, obvious enhancement of ECL signal was observed when luminol mixed with ZIF-67, and this signal was further enhanced after AuPt NPs introduced into the system. Hence, the synthesized nanozymes possessed superior catalytic performance attributed to ZIF-67 high peroxidase activity, which was consistent with the mechanism proposed above.

After luminol combined with AuPt/ZIF-67, the as-synthesized hybrids exhibited excellent catalytic performance with an obvious ECL signal boost Fig. 3B (curve a). When adding 20 mM GSH to the solution, a significant quenching of the signal could be observed (curve b), which was due to the consumption of oxygen free radicals in the system. However, after adding MnO_2_@CNTs quenchers to the substrate pool, the ECL signal further decreased though without GSH (curve c). The quenching mechanism may be that the absorption spectrum of MnO_2_ have proper overlaps with the ECL emission spectrum of luminol, which resulted in the quenching of ECL signal caused by ECL-RET [27]. Following the introduction of GSH in the luminol/H_2_O_2_ coreactants system, the ECL signal was obviously attenuated (curve d) compared with the MnO_2_@CNTs/luminol@AuPt/ZIF-67 modified electrode without GSH. The significant quenching effect mainly resulted from a large quantity of Mn^2+^ which was generated by GSH reduced MnO_2_, and then Mn^2+^ could consume the oxygen free radicals in the system and generate MnOO^+^ [40]. The ECL immunosensor was successfully established based on the above dual-quench strategy which included ECL-RET between MnO_2_ and luminol, as well as consumption of ROSs by Mn^2+^.

### Characterizations of the prepared ECL immunosensor

ECL dynamic curve and electrochemical impedance spectroscopy (EIS) were used to research the stepwise modification process of the ECL immunosensor. As displayed in Fig. 3C, when luminol@AuPt/ZIF-67 was decorated on the surface of GCE, the observed ECL signal was much higher than that of bare GCE (curve a via curve b), indicating that AuPt/ZIF-67 could boost the electron transfer to enhance ECL signal. With the following immobilization of Ab_1_ (curve c), BSA (curve d) and 10 ng mL^−1^ RBP4 (curve e), the ECL signal decreased continuously because the deposited insulating proteins impeded the electron transfer between luminophor and H_2_O_2_. Moreover, the ECL signal was decreased dramatically (curve f) when the modified electrode was incubated with Ab_2_-MnO_2_@CNTs bioconjugates. After introducing 20 mM GSH to the system, the ECL strength further diminish (curve g) which was in accord with the above hypothesis.

As shown in Fig. 3D, the impedance curves of stepwise modification process were consistent with ECL results. High frequency semicircle of bare GCE was very small (curve a), indicating faster electron transfer on the electrode surface. When luminol@AuPt/ZIF-67 were modified on the electrode surface of GCE, high frequency semicircle slightly increased (curve b) which was related to the low conductivity of chitosan and ZIF-67. After being modified with Ab_1_ (curve c), BSA (curve d), RBP4 (curve e) and Ab_2_-MnO_2_@CNTs (curve f), the resistance increased because of their negative charge that block the electron transfer rate. Since MnO_2_ could be consumed by GSH, thereby stripping the MnO_2_ from the Ab_2_-MnO_2_@CNTs bioconjugates, the semicircle domain was decreased (curve g). Cyclic voltammetry (CV) curves of stepwise modification process (Additional file 1: Fig. S5) were consistent with ECL and EIS results, indicating the successful fabrication of modified electrode as expected.

### Optimization of the experimental conditions

**Fig. 4:**
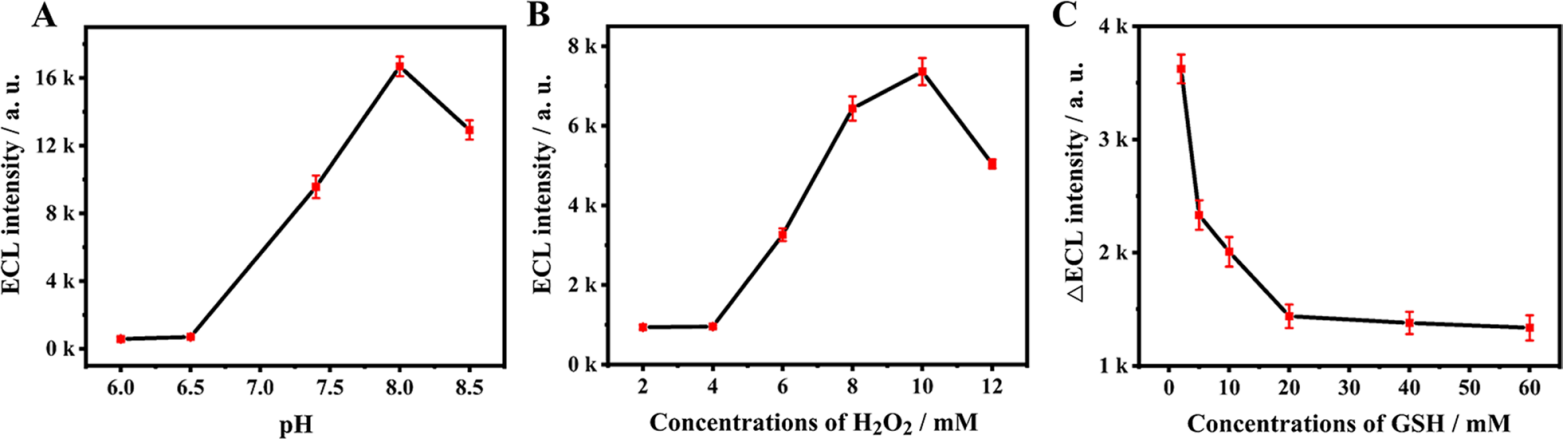
Optimization of the detection conditions. **A** The effect of pH on the response of ECL intensity from luminol@AuPt/ZIF-67/GCE; **B** the concentrations of H_2_O_2_ in luminol/H_2_O_2_ coreactants; **C** the effect of GSH concentrations on the ECL immunosensor for detection 5 ng mL^−1^ RBP4 (error bars, SD, n = 3)

To obtain excellent analytical capability of the prepared immunosensor, the experimental conditions of the experiment were systematically optimized. Luminol ECL luminescence effect highly relies on pH value of solution, and only exhibits high efficiency in alkaline medium. The ECL signal sharply increased with the increasing pH value from 6.0 to 8.0, and gradually decreased beyond pH 8.0 (Fig. 4A). Hence, the pH value selected in this experiment was 8.0. The concentration of coreactant H_2_O_2_ was optimized in Fig. 4B. When 10 mM H_2_O_2_ was introduced to the detection solution, the ECL signal declined visibly with the further increasing of H_2_O_2_. Therefore, the optimized concentration of H_2_O_2_ was 10 mM, which would be used in subsequent experiments. Furthermore, the amount of Mn^2+^ which was generated by GSH reduced MnO_2_ played a key role in the quenching effect of the ECL signal. After introducing 20 mM GSH to the detection solution, the △ECL intensity (difference between RBP4/BSA/Ab_1_/luminol@AuPt/ZIF-67/GCE and Ab_2_-MnO_2_@CNTs/RBP4/BSA/Ab_1_/luminol@AuPt/ZIF-67/GCE) reached a platform (Fig. 4C). Consequently, 20 mM GSH was selected for subsequent ECL detection to ensure optimal results.

The pulse potential also played an important role in ECL immunosensor and was optimized in the range 0.3–0.55 V. As shown in Additional file 1: Fig. S6A, the ECL signal reached its highest value at 0.45 V, which might be attributed to luminol can be electrochemically oxidized to luminol anion efficiently under a higher potential. The ECL signal showed an increasing to negative trend in the range − 0.6 to − 0.35 V in Additional file 1: Fig. S6B. The maximum signal was obtained at − 0.55 V, which may be because ECL initial potential of − 0.55 V has better diffusion control on the electrode surface.

### ECL detection performance of the immunosensor towards RBP4

**Fig. 5:**
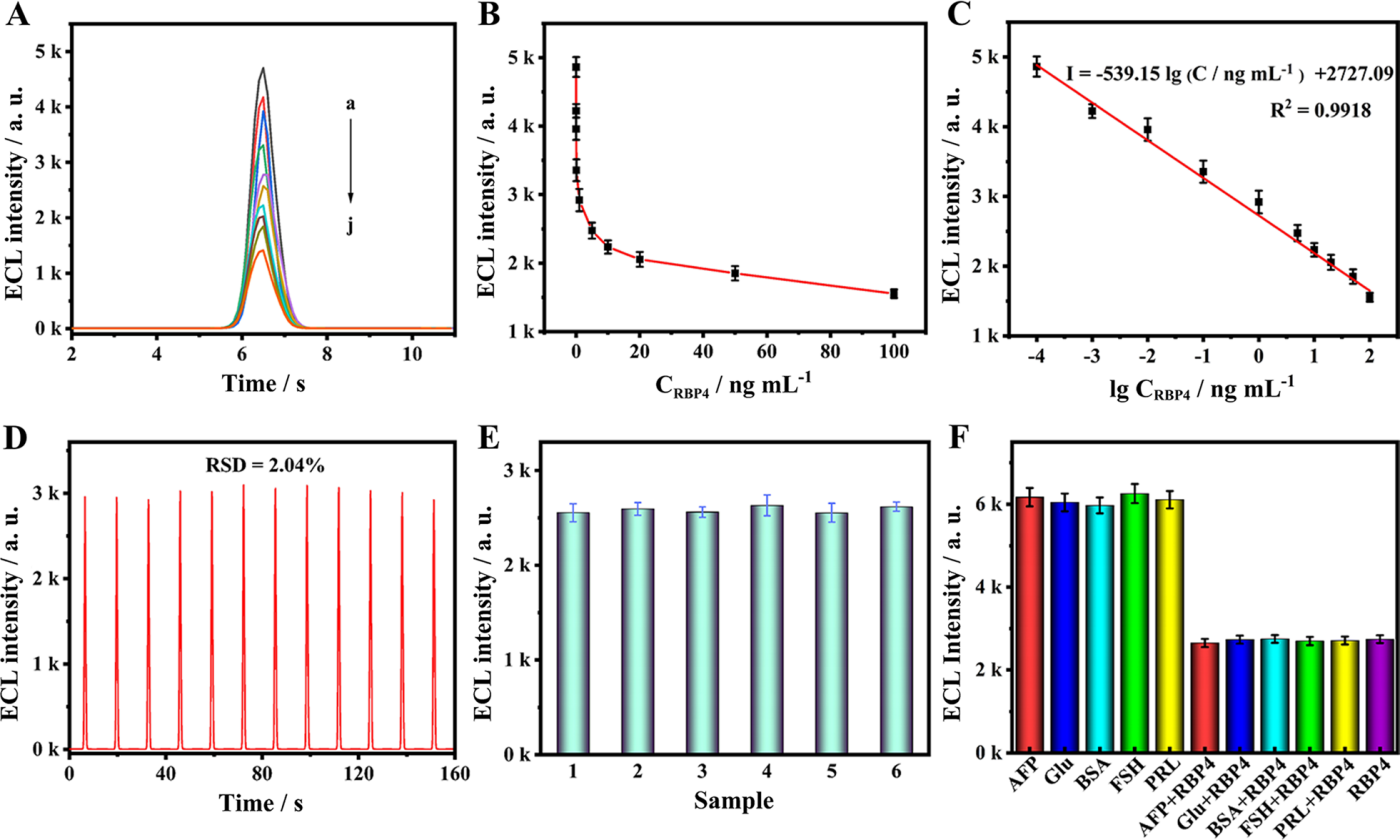
**A** The ECL intensities of the proposed ECL immunosensor incubated with different concentrations of RBP4 (0.0001, 0.001, 0.01, 0.1, 1, 5, 10, 20, 50, 100 ng mL^−1^); **B** correlation curve of ECL intensity as a function of RBP4; **C** calibration curve of the immunosensor; **D** the stability of the immunosensor for detecting 1 ng mL^−1^ RBP4; **E** reproducibility of the immunosensor incubated with 5 ng mL^−1^ RBP4 in PBS (pH = 8.0), **F** selectivity of the immunosensor against different interfering substances: AFP (10 ng mL^−1^), Glu (10 ng mL^−1^), BSA (10 ng mL^−1^), FSH (10 ng mL^−1^), PRL (10 ng mL^−1^) and 1 ng mL^−1^ RBP4 with 10 ng mL^−1^ interfering substances (error bars, SD, n = 3)

The efficacy of the prepared ECL immunosensor as a quantitative assay was investigated by varying the concentration of the RBP4 from 0.0001 to 100 ng mL^−1^ (curve a–j in Fig. 5A). As seen in the calibration plots of Fig. 5C, the correlation between the obtained ECL responses and the logarithm of the concentrations of RBP4 exhibited a good linear relationship in the range of 0.0001–100 ng mL^−1^. The linear equation was I = − 539.15 lg C + 2727.09 (where I was ECL intensity and C was the concentration of RBP4) with the correlation coefficient (R^2^) of 0.9918. The detection limit was calculated to be 43 fg mL^−1^ at a signal-to-noise ratio of 3 (S/N = 3). In addition, compared with previously reported RBP4 detection methods (Additional file 1: Table S1), our method showed higher sensitivity and lower detection limits, attributing to the high electrocatalytic activity of AuPt/ZIF-67 nanozyme and robust signal quenching ability of the tailored MnO_2_@CNTs.

### Specificity, reproducibility, and stability of the immunosensor

The specificity of the proposed immunosensor for RBP4 detection was evaluated to demonstrate its practicability. For this purpose, various substances such as alpha fetoprotein (AFP), follicle stimulating hormone (FSH), prolactin (PRL), bovine serum albumin (BSA) and glucose (Glu) were selected as potential interferences. The concentration of RBP4 was chosen as 1 ng mL^−1^, and all interfering substances as 10 ng mL^−1^, which was much higher than the concentration of RBP4 selected for detection in the system. As shown in Fig. 5F, only when RBP4 and its mixture existed, the ECL signal could decrease significantly, which indicated that the immunosensor possessed an excellent selective response to RBP4 against other relevant biomolecules.

Meanwhile, we also evaluated the reproducibility of the fabricated immunosensor. The reproducibility of this immunosensor was obtained under the same reaction condition by measuring 6 identical electrodes for detecting 5 ng mL^−1^ RBP4. The measured relative standard deviation (RSD) was 1.31% (Fig. 5E). To make the conclusions more credible, we have further optimized the reproducibility of the sensor at low (0.001 ng mL^−1^ RBP4), medium (5 ng mL^−1^ RBP4) and high concentrations (50 ng mL^−1^ RBP4), the measured RSD were 5.09%, 1.31% and 4.53% respectively (Additional file 1: Fig. S7), indicating good reproducibility of the proposed immunosensor.

The stability of the immunosensor was an important factor in evaluating whether it can be promoted in clinical practice. As illustrated in Fig. 5D, a steady ECL signal for RBP4 (1 ng mL^−1^) within 160 s was observed with the average RSD of 2.04%, indicating that the prepared immunosensor possessed excellent stability. Additionally, the ECL stability of the luminol@AuPt/ZIF-67/GCE was investigated in Additional file 1: Fig. S8, with the RSD of 0.14%, which demonstrated that the sensing signal was reliable. Meanwhile, the proposed immunosensor was investigated by recording the ECL behavior storing for 10 days at 4 °C. The ECL intensity decreased by 15.7% compared with the original ECL intensity after 10 days (Additional file 1: Fig. S9). These results suggested that the stability of the biosensor was satisfactory.

### Clinical sample analysis of the immunosensor

To estimate the recovery efficiency of the immunosensor in complex serum matrices, the standard spike-and-recovery experiment was carried out. As shown in Table 1, the ECL immunosensor could be applied to detecting RBP4 in real human serum samples with a recovery of 96.60–111.32%. All these results demonstrated that the proposed ECL immunoassay had potential applications in potentially detecting biomarkers of T2DM in serum.

**Table 1:** The recoveries of retinol binding protein 4 using the proposed ECL modified electrodes measured in 5 mL PBS (0.1 M, pH = 8.0)

Sample	Addition (ng mL^−1^)	ECL intensity (a. u.)	Found (ng mL^−1^)	RSD (%, n = 3)	Recovery (%, n = 3)
1	50.00	1811.09	50.91	3.49	101.82
2	25.00	1973.39	25.09	4.26	100.36
3	5.00	2350.24	4.85	4.92	97.00
4	0.50	2889.39	0.5566	2.74	111.32
5	0.05	3428.54	0.0483	5.21	96.60

## Conclusions

In summary, based on luminol@AuPt/ZIF-67 hybrids and MnO_2_@CNTs composites, a dual-quenched ECL-RET immunosensor for ultrasensitive detection of RBP4 has been successfully constructed. The multi-function AuPt/ZIF-67 hybrids were employed as efficient peroxidase-like nanozymes, ECL donors and nanocarriers to load abundant luminol and antibodies, thus boosting the ECL performance of the luminol-H_2_O_2_ system. Subsequently, the amplified initial signal can be effectively quenched by MnO_2_@CNTs composites through inhibiting peroxidase-like activity and ECL-RET strategy of luminol@AuPt/ZIF-67. Taking advantage of this dual signal quenching between luminol@AuPt/ZIF-67 hybrids and MnO_2_@CNTs composites, the proposed ECL immunosensor for RBP4 detection achieved remarkable sensitivity, specificity and stability. In addition, the designed ECL immunosensor provides a promising platform not only for ultrasensitive detection of RBP4 to early monitor and diagnose T2DM, but also other biomarkers analysis. Obviously, future studies need to focus on simplifying detection procedure to promote clinical applications of ECL technique.

## Supplementary Information

**Additional file 1. Fig. S1.** XRD pattern of Simulated ZIF-67, pure ZIF-67 and AuPt/ZIF-67. **Fig. S2.** XPS spectra of the MnO2@CNTs. **Fig. S3.** Typical catalytic oxidation reaction of 3, 3′, 5, 5′-tetramethylbenzidine. **Fig. S4.** UV/Vis absorption spectra of ZIF-67, luminol, AuPt/ZIF-67 and luminol@AuPt/ZIF-67. **Fig. S5.** Cyclic voltammetry (CV) curves of stepwise modification process. **Fig. S6.** Optimization of the reaction conditions. Reproducibility and stability of the immunosensor (**Fig. S7, Fig. S8** and **Fig. S9.**). **Table S1.** Comparison of ECL Immunosensor with other reported method.

## Data Availability

The datasets used and/or analysed during the current study are available from the corresponding author on reasonable request.
