# Supplementary material for: A dual-quenched ECL immunosensor for ultrasensitive detection of retinol binding protein 4 based on luminol@AuPt/ZIF-67 and MnO2@CNTs
Source: J Nanobiotechnology. 2021 Sep 8;19:272. doi: 10.1186/s12951-021-01020-1 (PMC8425071; doi:10.1186/s12951-021-01020-1)
Supplement: Supplementary file 1 — Additional file 1. Fig. S1. XRD pattern of Simulated ZIF-67, pure ZIF-67 and AuPt/ZIF-67. Fig. S2. XPS spectra of the MnO2@CNTs. Fig. S3. Typical catalytic oxidation reaction of 3, 3′, 5, 5′-tetramethylbenzidine. Fig. S4. UV/Vis absorption spectra of ZIF-67, luminol, AuPt/ZIF-67 and luminol@AuPt/ZIF-67. Fig. S5. Cyclic voltammetry (CV) curves of stepwise modification process. Fig. S6. Optimization of the reaction conditions. Reproducibility and stability of the immunosensor (Fig. S7, Fig. S8 and Fig. S9.). Table S1. Comparison of ECL Immunosensor with other reported method. [file 12951_2021_1020_MOESM1_ESM.docx]

Additional file 1

A dual-quenched ECL immunosensor for ultrasensitive detection of retinol binding protein 4 based on luminol@AuPt/ZIF-67 and MnO_2_@CNTs

Wei Gong^1,2†^, Suqing Yang^3†^, Fen Zhang^1,2^, Fengshun Tian^1,2^, Junman Chen^4^, Zhigang Yin^4^, Shijia Ding^4^, Wei Yang^4,*^, Rong Luo^1,2,^^[[1]](#footnote-1)^*

^1^ *Medical Examination Centre, The First Affiliated Hospital of Chongqing Medical University, Chongqing 400016, China*

^2^ *Department of Endocrinology, The First Affiliated Hospital of Chongqing Medical University, Chongqing 400016, China*

^3^ *Chongqing Testing & lnspection Center for Medical Devices, Chongqing 400016, China*

^4^ *Key Laboratory of Clinical Laboratory Diagnostics (Ministry of Education), College of Laboratory Medicine, Chongqing Medical University, Chongqing, 400016, China*

**Reagents and Materials**

Luminol, 2-methylimidazole (MeIM), Cobalt nitrate hexahydrate (Co(NO_3_)_2_•6H_2_O), Chloroauric acid hexahydrate (HAuCl_4_•6H_2_O), Chloroplatinic acid hexahydrate (H_2_PtCl_6_•6H_2_O), Sodium borohydride (NaBH_4_), Carbon nanotubes (CNTs), Potassium permanganate (KMnO_4_), Bovine serum albumin (BSA, 96-99%), Poly dimethyl diallyl ammonium chloride (PDDA) and Chitosan were all supplied from Sigma-Aldrich. Human Retinol Binding Protein 4 (RBP4) (ab59967), Retinol Binding Protein 4 monoclonal antibody (RBP4 Ab_1_) (ab109193) and Goat Anti-Rabbit IgG H&L (RBP4 Ab_2_) (ab205718) were obtained from Abcam USA. Methanol, Ethanol and sulfuric acid (H_2_SO_4_) were purchased from Kelong Chemical Inc. (Chengdu, China).

**Apparatus and Measurements**

ECL measurements were used by a MPI-E capillary electrophoresis electrochemiluminescence detector (Xi’an Remax Analysis Instruments Co. Ltd., China) and electrochemical measurements were performed on CHI660D electrochemical workstation (Shanghai Chenhua Instruments Co. Ltd., China) with a three-electrode system composed of a platinum wire (auxiliary electrode), a Ag/AgCl electrode (reference electrode) and a glassy carbon electrode (GCE, *Φ* = 3 mm) (working electrode). Transmission electron microscopy (TEM), High-angle annular dark-field (HAADF) and Scanning transmission electron microscopy-energy dispersive X-ray spectroscopy (STEM-EDS) were recorded by Tecnai G2 F30 S-TWIN microscope (USA). X-ray photoelectron spectroscopy (XPS) spectra were registered with an Thermo ESCALAB 250Xi (USA). X-ray diffraction (XRD) patterns of the prepared samples were gained with a D8 ADVANCE X-ray diffractometer (Bruker, Germany) to confirm the structure of the materials. The ultraviolet-visible (UV-vis) spectrophotometer (UV-2450, Shimadzu, Kyoto, Japan) was used to demonstrate features of the nanomaterials. The ECL measurement was performed in 5 mL PBS (0.1 M, pH 8.0) containing 10 mM H_2_O_2_ under potential scanning from -0.55 to 0.45 V at a scan rate of 0.15 V s^-1^, and the photomultiplier tube voltage selected at 400 V.





**Fig.** **S1** XRD pattern of Simulated ZIF-67, pure ZIF-67 and AuPt/ZIF-67.

**
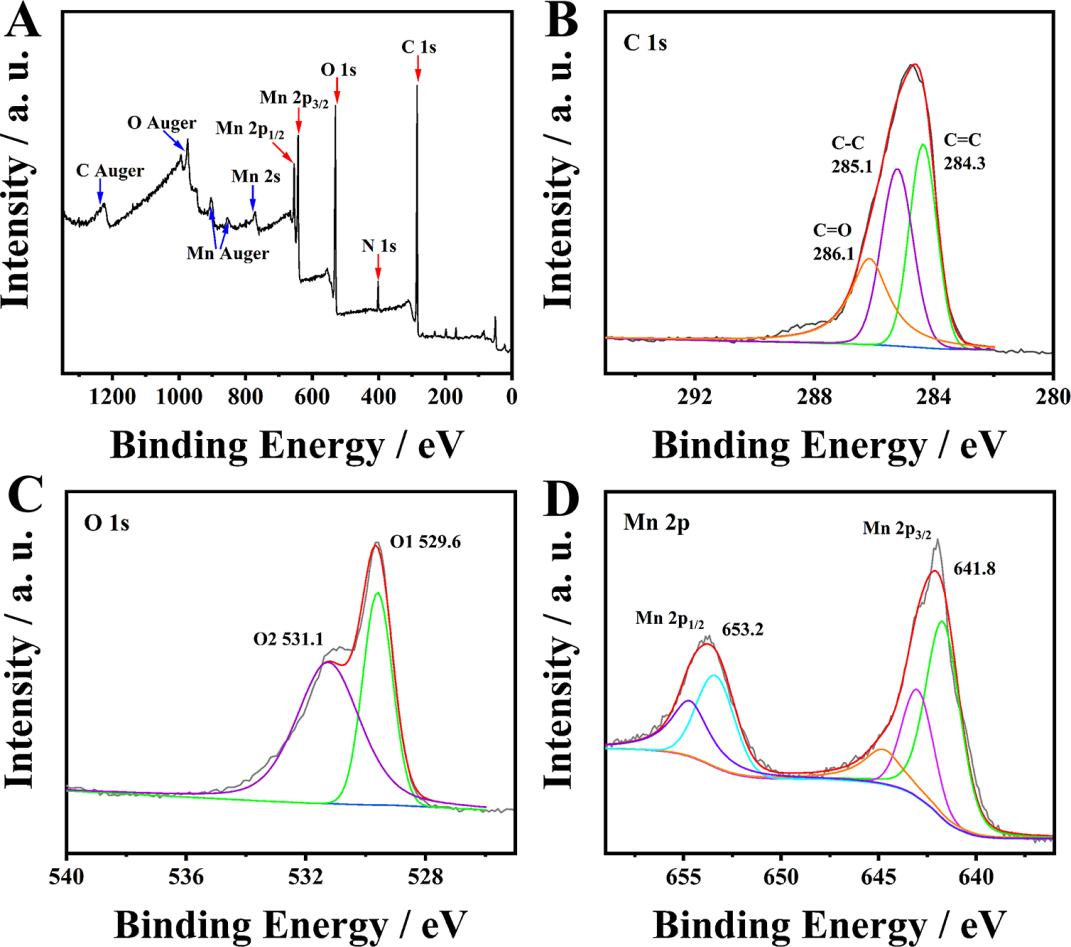
**

**Fig. S2** XPS spectra of the MnO_2_@CNTs composites: (A) full spectrum; (B) C 1s; (C) O 1s; (D) Mn 2p.

**Typical catalytic oxidation reaction of 3, 3′, 5, 5′-tetramethylbenzidine**

A typical catalytic oxidation reaction of the peroxidase substrate 3, 3′, 5, 5′-tetramethylbenzidine (TMB) by H_2_O_2_ was performed to investigate the catalytic activity of CNTs and ZIF-67. As shown in Fig. S2, only after ZIF-67 suspension was added to the mixture of TMB and H_2_O_2_, the colorless solution (tube b) immediately turned bright blue color, while the CNTs (tube a) did not show a TMB chromogenic reaction in the presence of H_2_O_2_. All these evidences suggested that the CNTs we used did not have any effect on the performance of the sensing.


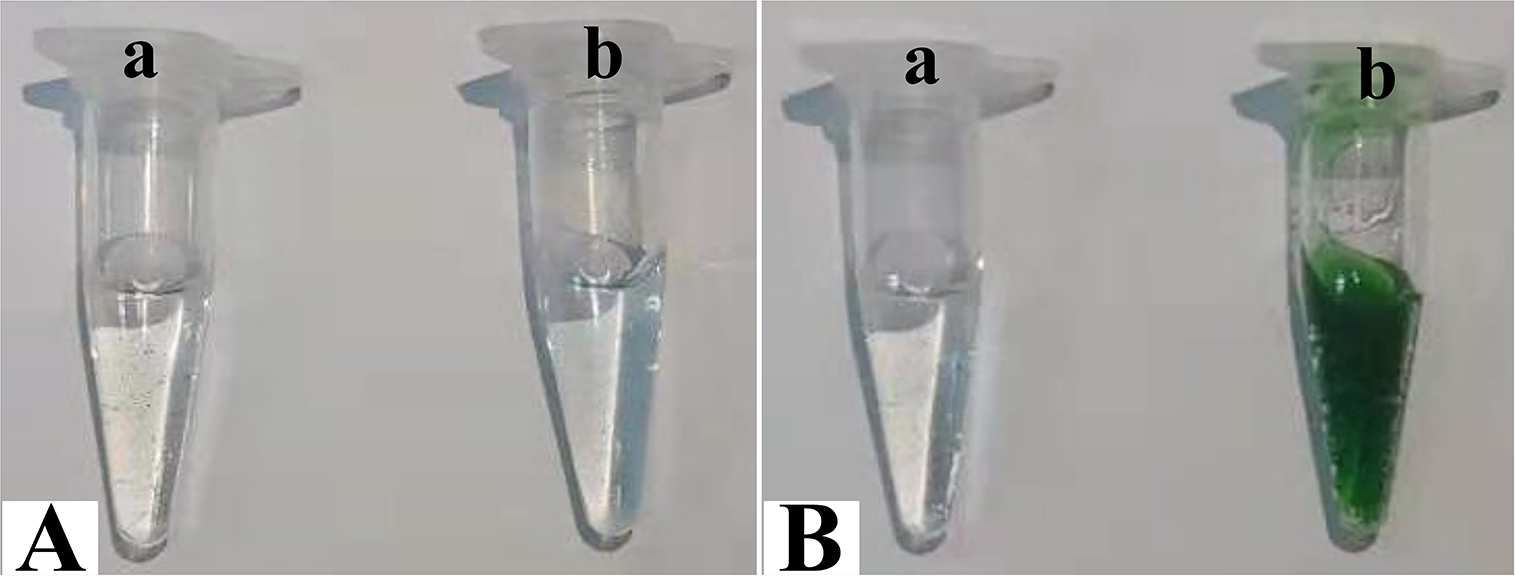


**Fig. S3** The image of TMB color reaction before (A) and after (B) H_2_O_2_ addition (a: CNTs; b: ZIF-67).





**Fig.** **S4** UV/Vis absorption spectra of (a) ZIF-67, (b) luminol, (c) AuPt/ZIF-67 and (d) luminol@AuPt/ZIF-67.





**Fig. S5** CV curve of bare GCE (a), after luminol@AuPt/ZIF-67 modification (b), after Ab_1_ modification (c), after BSA blocking (d), after 10 ng mL^-1^ RBP4 incubation (e), after Ab_2_-MnO_2_@CNTs immobilization (f), and after adding 20 mM GSH (g), measured in 5 mM [Fe(CN)_6_]^3-/4-^ containing 0.1 M KCl at 150 mV/s.

**
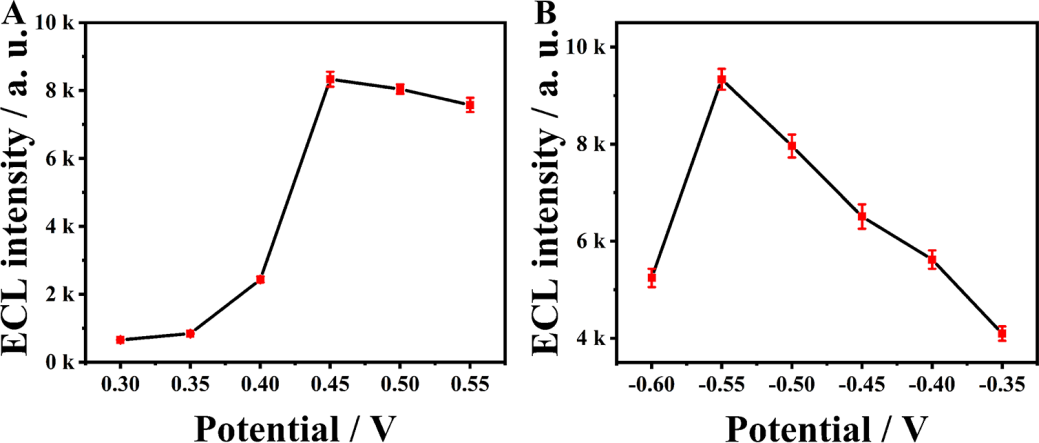
**

**Fig. S6** Effect of pulse amplitude on the response signal in the solution of 10 mM H_2_O_2_ in 0.1 M PBS (pH 8.0) buffer, PMT 400 V (Error bars, SD, n=3).


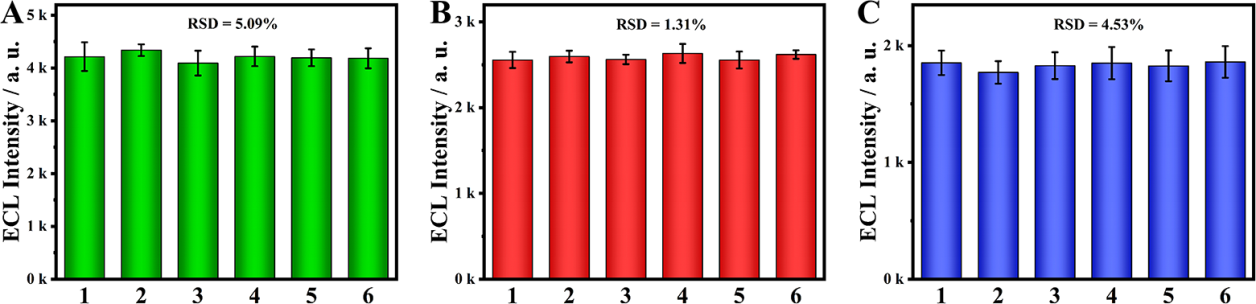


**Fig. S7** Reproducibility of the immunosensor incubated with 0.001 ng mL^-1^ RBP4 (A), 5 ng mL^-1^ RBP4 (B), and 50 ng mL^-1^ RBP4 (C) in PBS (pH = 8.0).





**Fig. S8** The stability of luminol@AuPt/ZIF-67/GCE in pH 8.0 PBS containing 10 mM H_2_O_2_.





**Fig. S9** Storage stability of the prepared immunosensor incubated with 5 ng mL^-1^ RBP4 and performed in pH 8.0 PBS containing 10 mM H_2_O_2_ (Error bars, SD, n=3).

**Table S1** Comparison of ECL Immunosensor with other reported method for determination of RBP4.

| **Detecting Method** | **Linear range** | **Detection limit** | **References** |
| --- | --- | --- | --- |
| ELAAS | 0.2-5 µg mL^-1^ | 78 ng mL^-1^ | [1] |
| ELISA | 1-3 ng mL^-1^ | > 10 ng mL^-1^ | [2] |
| SPR | 0.075-2 ng mL^-1^ | 75 pg mL^-1^ | [3] |
| DNA immunization | 0.1-20 ng mL^-1^ | 1 ng mL^-1^ | [4] |
| Electrochemical sensor | 0.0001-1 ng mL^-1^ | 0.1 pg mL^-1^ | [5] |
| ECL | 0.001-100 ng mL^-1^ | 43 fg mL^-1^ | This work |

ECL: electrochemiluminescence; ELISA: enzyme linked immunosorbent assay; SPR: Surface Plasmon Resonance

**References**

[1] S.J. Lee, J.-W. Park, I.-A. Kim, B.-S. Youn, M.B. Gu, Sensitive detection of adipokines for early diagnosis of type 2 diabetes using enzyme-linked antibody-aptamer sandwich (ELAAS) assays, Sens. Actuators B Chem. 2012; 168: 243-248.

[2] N.S. Lee, H.S. Kim, S.E. Park, M. Bluher, C.Y. Park, B.S. Youn, Development of a mouse IgA monoclonal antibody-based enzyme-linked immunosorbent sandwich assay for the analyses of RBP4, Sci. Rep. 2018; 8 (1): 2578.

[3] S.J. Lee, B.-S. Youn, J.W. Park, J.H. Niazi, Y.S. Kim, M.B. Gu, ssDNA Aptamer-Based Surface Plasmon Resonance Biosensor for the Detection of Retinol Binding Protein 4 for the Early Diagnosis of Type 2 Diabetes, Anal. Chem. 2008; 80 (8): 2867-2873.

[4] C. Bian, F. Zhang, F. Wang, Z. Ling, M. Luo, H. Wu, Y. Sun, J. Li, B. Li, J. Zhu, L. Tang, Y. Zhou, Q. Shi, Y. Ji, L. Tian, G. Lin, Y. Fan, N. Wang, B. Sun, Development of retinol-binding protein 4 immunocolloidal gold fast test strip using high-sensitivity monoclonal antibodies generated by DNA immunization, Acta Biochim. Biophys. Sin. (Shanghai) 2010; 42 (12): 847-53.

[5] A. Paul, M.S. Chiriacò, E. Primiceri, D.N. Srivastava, G. Maruccio, Picomolar detection of retinol binding protein 4 for early management of type II diabetes, Biosens. Bioelectron. 2019; 128: 122-128.

1. *Correspondence: E-mail address: luorongy@163.com; yang623621810@163.com

   ^†^ Wei Gong and Suqing Yang contributed equally to this manuscript [↑](#footnote-ref-1)
